# Comparative genetic analysis of a rare synchronous collision tumor composed of malignant pleural mesothelioma and primary pulmonary adenocarcinoma

**DOI:** 10.1186/s13000-016-0488-0

**Published:** 2016-04-18

**Authors:** Tomoaki Naka, Yutaka Hatanaka, Katsuji Marukawa, Hiromi Okada, Kanako C. Hatanaka, Jun Sakakibara-Konishi, Satoshi Oizumi, Yasuhiro Hida, Kichizo Kaga, Tomoko Mitsuhashi, Yoshihiro Matsuno

**Affiliations:** Department of Surgical Pathology, Hokkaido University Hospital, Kita 14, Nishi 5, Kita-ku, Sapporo, Hokkaido 060-8648 Japan; Research Division of Companion Diagnostics, Hokkaido University Hospital, Kita 14, Nishi 5, Kita-ku, Sapporo, Hokkaido 060-8648 Japan; First Department of Medicine, Hokkaido University School of Medicine, Kita 15, Nishi 7, Kita-ku, Sapporo, Hokkaido 060-8638 Japan; Department of Cardiovascular and Thoracic Surgery, Graduate School of Medicine, Hokkaido University, Kita 15, Nishi 7, Kita-ku, Sapporo, Hokkaido 060-8638 Japan

**Keywords:** Copy number alteration, Collision tumor, Malignant pleural mesothelioma

## Abstract

**Background:**

Although asbestos acts as a potent carcinogen in pleural mesothelial and pulmonary epithelial cells, it still remains unclear whether asbestos causes specific and characteristic gene alterations in these different kinds of target cells, because direct comparison in an identical patient is not feasible. We experienced a rare synchronous collision tumor composed of malignant pleural mesothelioma (MPM) and primary pulmonary adenocarcinoma (PAC) in a 77-year-old man with a history of long-term smoking and asbestos exposure, and compared the DNA copy number alteration (CNA) and somatic mutation in these two independent tumors.

**Methods:**

Formalin-fixed paraffin-embedded (FFPE) tissues of MPM and PAC lesions from the surgically resected specimen were used. Each of these MPM and PAC lesions exhibited a typical histology and immunophenotype. CNA analysis using SNP array was performed using the Illumina Human Omni Express-12_FFPE (Illumina, San Diego, CA, USA) with DNA extracts from each lesion. Somatic mutation analysis using next-generation sequencing was performed using the TruSeq Amplicon Cancer Panel (Illumina).

**Results:**

The CNA analysis demonstrated a marked difference in the frequency of gain and loss between MPM and PAC. In PAC, copy number (CN) gain was detected more frequently and widely than CN loss, whereas in MPM there was no such obvious difference. PAC did not harbor CNAs that have been identified in asbestos-associated lung cancer, but did harbor some of the CNAs associated with smoking. MPM exhibited CN loss at 9p21.2-3, which is the most common genetic alteration in mesothelioma.

**Conclusion:**

In this particular case, asbestos exposure may not have played a primary role in PAC carcinogenesis, but cigarette smoking may have contributed more to the occurrence of CN gains in PAC. This comparative genetic analysis of two different lesions with same amount of asbestos exposure and cigarette smoke exposure has provided information on differences in the cancer genome related to carcinogenesis.

## Background

Asbestos is well known to be a causative agent both of malignant mesothelioma and lung cancer. It has been reported that asbestos causes genetic alterations at the chromosomal level [1]. Loss at 9p21 and DNA copy number alteration (CNA) have been identified in mesothelioma [2–6], whereas allelic imbalance at 2p16, 9q33.1 and 19p13 has been reported in asbestos-associated lung cancer [7]. Although asbestos is considered to act as a potent carcinogen both in pleural mesothelial cells and pulmonary epithelial cells, several studies have suggested that there seems to be less analogous abnormality observed between neoplasms derived from each of them [8]. However, it still remains unclear whether asbestos causes specific and characteristic gene alterations in these different kinds of target cells, because simultaneous occurrence of these neoplasms after asbestos exposure in an identical patient is exceedingly rare [9, 10], and thus direct comparison of the respective gene alterations is not feasible. We encountered a patient with a history of both long-term smoking and asbestos exposure who underwent extrapleural pneumonectomy and was proved to have a synchronous collision tumor consisting of malignant pleural mesothelioma (MPM) and primary pulmonary adenocarcinoma (PAC). It was expected that comprehensive genetic analysis of these two different tumors with a possible relationship to asbestos exposure and cigarette smoke exposure would aid understanding of thoracic carcinogenesis. In the present study, therefore, we compared CNA and somatic mutation in this case synchronous collision tumor consisting of MPM and PAC.

## Case presentation

### Clinical summary

A 77-year-old Japanese man with a 20-pack-year history of cigarette smoking also had a history of asbestos exposure while working in the construction industry between the ages of 40 and 65. He was admitted to our hospital complaining of coughing, sputum and breathlessness, which had developed gradually. A chest X-ray of his left lung demonstrated an abnormal shadow, and chest computed tomography (CT) revealed left pleural effusion, diffuse pleural thickening and infiltrates with cavitations in the left lung. The levels of all serum tumor markers examined, including carcinoembryonic antigen (CEA), carbohydrate antigen 19-9 (CA19-9), squamous cell carcinoma antigen (SCC), neuron-specific enolase (NSE), cytokeratin fragment (CYFRA), and pro-gastrin-releasing peptide (Pro-GRP), were within the normal ranges. Cytological examination of the left pleural effusion detected malignant mesothelial cells, and a preoperative clinical diagnosis of MPM was made. Chest and abdominal CT imaging demonstrated no detectable distant metastasis. Left extrapleural pneumonectomy was performed.

### Pathologic findings

**Fig. 1:**
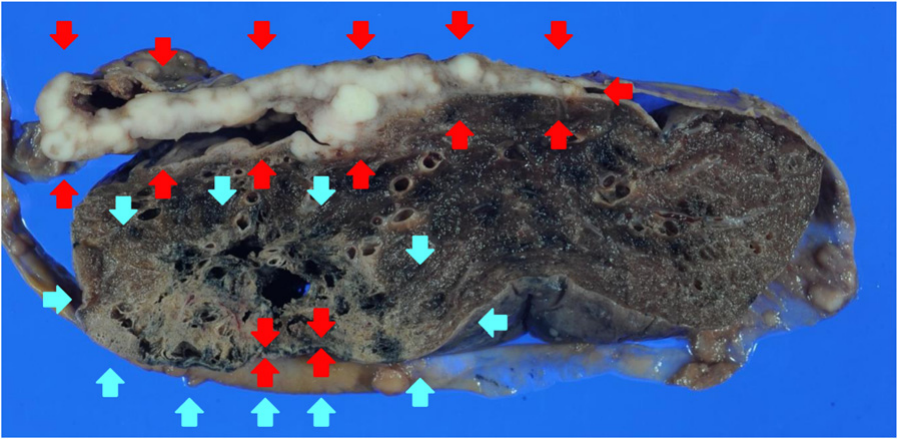
Macroscopic appearance of the synchronous collision tumor. A cross-section of the resected specimen shows diffuse pleural thickening confluent with the multinodular grayish-white solid tumor (*red arrow*), and an intrapulmonary tumor showing an irregular and ill-defined border and a gray-white cut surface (*blue arrow*) colliding within the left lower lobe of the lung

Cross-sectional examination of the resected specimen showed that the left lung was widely covered by diffuse pleural thickening confluent with the multinodular grayish-white solid tumor (Fig. 1). The tumor involved both the parietal and visceral pleurae, invading the diaphragm and mediastinal tissues surrounding the thoracic aorta. In the close vicinity of this major pleural tumor described above, an intrapulmonary tumor was found in the lower lobe (Fig. 1). This latter tumor had an ill-defined border and a gray-white cut surface. This intrapulmonary tumor had not been demonstrated in the preoperative imaging work-up.

**Fig. 2:**
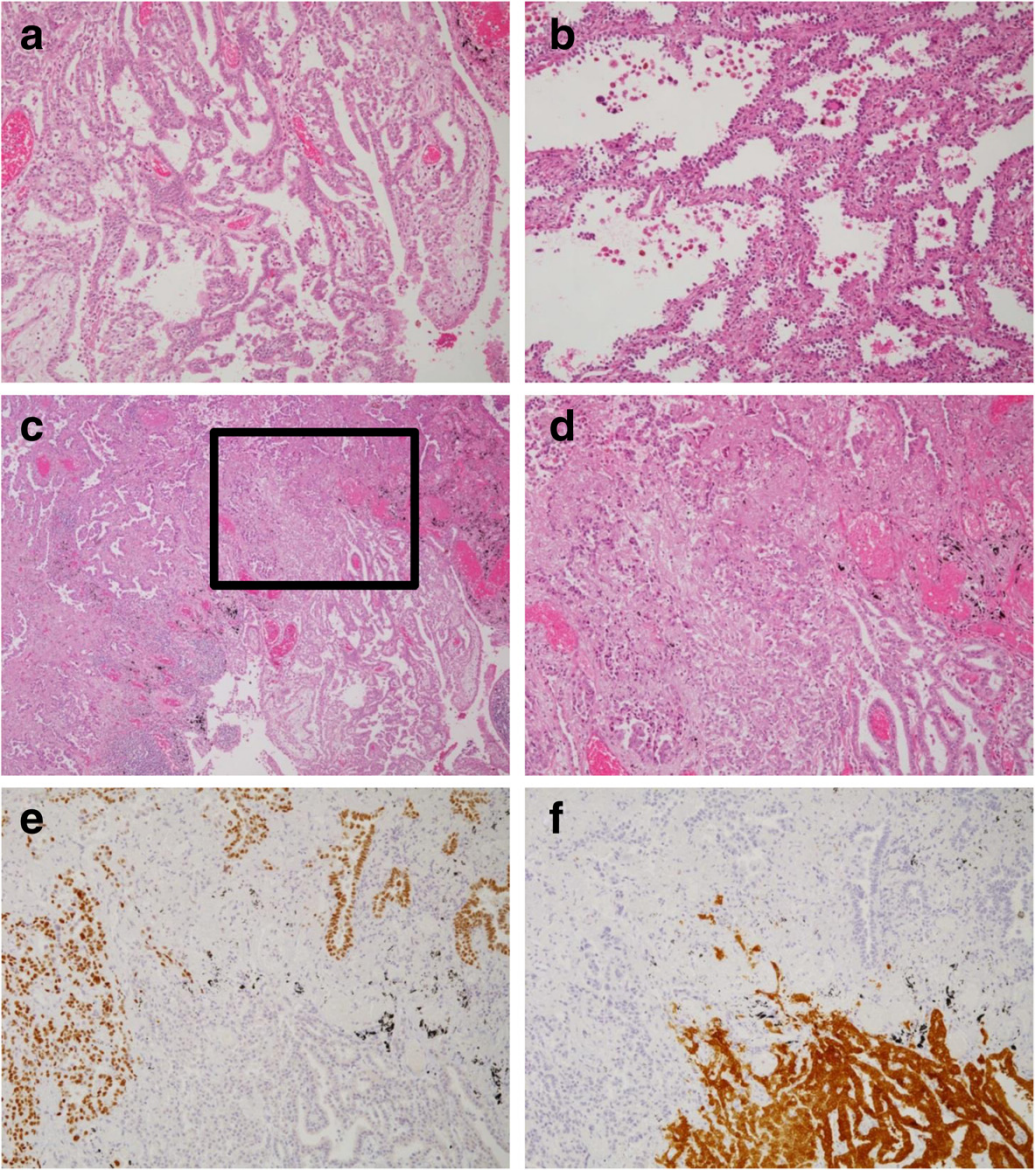
Histology and immunohistochemistry of the two lesions of the synchronous collision tumor. Representative histologic features of the diffuse pleural thickening (**a**), epithelioid-type MPM, and the intrapulmonary tumor (**b**), PAC. **c** and **d** show the area of collision, PAC being distributed on the upper left, and MPM on the lower right. **d** Is the magnified images of the rectangle area in (**c**). MPM and PAC exhibit typical immunohistochemical staining for TTF-1 (**e**) and calretinin (**f**), respectively

Histologically, these two tumors showed distinct features. The pleural tumor was composed of neoplastic cells growing in a tubulopapillary pattern, or in solid sheets or nests in some areas (Fig. 2a). Most of the papillae were covered by a single layer of cuboidal tumor cells, and the pseudoglands were lined by similar cells, often with a solid growth pattern. These histologic findings were conclusive of MPM, epithelioid type. On the other hand, the intrapulmonary tumor was composed of neoplastic cells showing lepidic growth in the peripheral portion, and papillary or acinar invasive growth patterns in the central portion (Fig. 2b). Its stroma varied from fibrous to desmoplastic. These findings suggested a diagnosis of PAC, which is invasive adenocarcinoma, lepidic predominant (lepidic 60 %, papillary 30 %, acinar 10 %) according to Sica’s classification [11]. The two tumors, MPM and PAC, collided within the same lower lobe of the left lung (Fig. 2c, d). Asbestos bodies were detected rather easily in HE-stained sections of the subpleural or peribronchial non-tumorous lung parenchyma.

Immunohistochemistry (IHC) showed that the cells in the pleural tumor were positive for calretinin and D2-40, but negative for TTF-1 and Ber-EP4 (Fig. 2e, f), being consistent with the diagnosis of epithelioid-type MPM. In contrast, the cells in the intrapulmonary tumor were positive for TTF-1 and Ber-EP4, but negative for calretinin and D2-40 (Fig. 2e, f), thus confirming that the latter tumor was PAC.

The results of our various examinations allowed us to make a final diagnosis of collision tumor consisting of two lesions: epithelioid-type MPM, and PAC. Molecular testing performed on the PAC showed negativity for EGFR, KRAS/NRAS/BRAF gene mutations and ALK fusion protein.

## Methods

### DNA extraction from formalin-fixed paraffin-embedded (FFPE) tissues

FFPE tissues of MPM and PAC were manual macrodissected and DNA was extracted with a QIAamp DNA FFPE Tissue Kit (Qiagen). The quantity of the purified DNA sample was analyzed with a PicoGreen dsDNA Quantitation Kit (Life Technologies, Carlsbad, CA, USA). The total DNA yield was >1000 ng for both MPM and PAC. The quality of the DNA sample was then assessed using a quantitative PCR assay with the Infinium HD FFPE QC kit (Illumina, San Diego, CA, USA) to determine if the DNA sample obtained was applicable to genetic analyses.

### CNA analysis using SNP array

After passing the DNA quality assessment, the sample with amplifiable DNA from FFPE tissues was then restored using the Infinium HD FFPE Restore Kit (Illumina) in accordance with the manufacturer’s instructions. The restored DNA sample was fluorescently labeled with cyanine dye Cy5, and control DNA was labeled with cyanine dye Cy3 in accordance with the manufacturer’s instructions. Labeled products were cohybridized to the Illumina Human Omni Express-12_FFPE (Illumina). Microarray data were analyzed using Genome Studio software (Illumina).

### Somatic mutation analysis using next-generation sequencing

Target sequencing was performed on the MiSeq platform using a TruSeq Amplicon Cancer Panel, which is a highly multiplexed next-generation sequencing (NGS) system covering 212 regions in 48 cancer-related genes (Illumina), and then the NGS data were analyzed using the MiSeq Reporter software [12].

## Results

### Genetic alteration in the MPM lesion

**Fig. 3:**
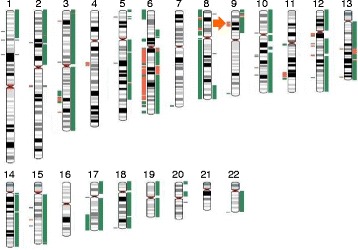
Karyotype of the synchronous collision tumor comparing MPM and PAC. Lines to the left of the chromosomes represent MPM and lines to the right represent PAC. Orange lines represent losses, green lines represent gains, and gray lines represent copy-neutral loss of heterozygosity (LOH). Loss of 9p21, which is the common genetic alterations in mesothelioma, was found in the MPM lesion (*orange arrow*)

In the MPM lesion, copy number (CN) gain was detected widely throughout almost the whole of chromosome 8 (Fig. 3). In addition, CN loss was detected in several limited regions in the long arm of chromosome 6 and at 9p21.2-3, both of which are the common genetic alterations in mesothelioma [2–6, 13]. Other short-region gains were found at 3q22-23, 9q34.2, 17q22-25, and 22q13.3, and losses were found at 1p31.2-1p12.1, 3p24.3, 4q21.13-22.1, 13q21.31, 13q33.3, and 15q22.2. Copy-neutral loss of heterozygosity (LOH) was found at 1p31.1, 2p16.2-16.1, 3p12.1, 5q14.3, 5p15.2, and 15q23-24.1. Gene alterations at 22q12.2 and 17p13.1 were not detected. Somatic mutation was observed in *ATM* (G2706A). Possible germline mutations of *TP53* (P72R) and *KDR* (Q472H) were also observed in both MPM and PAC lesions with mutation rates of almost 50 % (data not shown).

### Genetic alteration in the PAC lesion

In the PAC lesion, CN gain occurred more frequently than loss (Fig. 3), and was found throughout almost the whole of the chromosomes 3, 10, 12, 17, 18, and 19, on the long arm of chromosomes 13, 14, 15, and 22, and on the short arm of chromosome 9. Other region gains were found at 1p36, 2p23-14,5p15.2, 5q21, 6q21-22.1, 8q24. Loss and copy-neutral LOH were rarely found. There were no detectable alterations at 2p16, 9q33.1 or 19p13, which have been reported previously in asbestos-associated lung cancer [7]. PAC had somatic mutation of *ERBB4* (300 fs, 301 fs) and *STK11* (E65G), as well as *TP53* (P72R) and *KDR* (Q472H) with the possibility of the germline mutations.

### Comparison of CNA between the MPM and PAC lesions

Our CNA analysis revealed that the frequency of gain and loss differed between MPM and PAC. In PAC, CN gain was frequently and widely detected in comparison with CN loss, whereas in MPM there was no such tendency for marked gain/loss imbalance, and large-region CNA was detected only on limited chromosomes, including chromosome 8 with CN gains. When we focused on individual chromosomes, CNAs were found in almost all regions of the 6q arm in both of the tumor lesions. No CNA was found on chromosome 16 or 21 in either MPM or PAC. Gains or losses on chromosomes 4, 7 and 11 were found only in MPM, and on chromosomes 14, 19 and 20 only in PAC.

## Discussion

Recent improvements in molecular-based technologies have enabled the use of routine FFPE tissue materials for highly informative genetic analyses. In the present study using these FFPE-related technologies, we attempted to analyze CNA and somatic mutation in a collision tumor consisting of MPM and PAC. Coexistence of MPM and PAC is extremely rare, although both may be caused by asbestos exposure. Only a few such cases have been reported previously, as judged from citations confirmable on the PubMed database [9, 10], and therefore we consider that this is the first reported study to have comprehensively compared CNA and somatic mutation in the MPM and PAC lesions of a collision tumor. We consider that comparative analysis of independent tumors occurring in a single patient would provide useful information on direct differences in the cancer genome without any biological variations in the tumor or the need to consider research cohort sizes.

In the present case, loss of 9p21.2-3 was detected in the MPM lesion. Deletion of 9p21, encoding the CDK inhibitors, p16^INK4a^, p14^ARF^ and p15^INK4b^, has been observed in up to 80 % of mesotheliomas [2–6]. Thus, in addition to the typical morphologic features, the MPM lesion of the present collision tumor harbored the gene alterations typical of malignant mesothelioma. Allelic loss of NF2, located at 22q12.2, has been reported in up to 70 % of mesotheliomas [2, 14, 15]. Loss of chromosome 17p13.1, encoding the p53 gene TP53, is also a common and important change in mesothelioma [2]. Gene alterations at 22q12.2 and 17p13.1 were not detected in the present case.

On the other hand, loss at 2p16 and 19p13, and gain and loss at 9q33.1 were recently identified in asbestos-associated lung cancer and considered to be associated with the genotoxic effect of asbestos [7]. The region encoding p16^INK4a^ has also been shown to have a higher incidence of LOH and homozygous deletion in asbestos-associated lung cancer [16]. In the present case, however, no alterations in these major regions were found in the PAC lesion. Conversely, CNA occurred widely in almost all chromosome regions and was more frequent than in the MPM lesion. These results suggest that asbestos exposure may not have played a primary role in PAC carcinogenesis in this particular case, and that factors other than asbestos may have been involved.

As mentioned above, this patient also had a 20-pack-year history of cigarette smoking in addition to asbestos exposure. As CNAs associated with smoking, 12q23, 3q24, 8q24, 5q, 8q, 16p, 19p, 22q, 15q25, 8p11.23-8p12, and 17p13.1 have been reported [17–21], and some of them were found in the PAC lesion in the present case. However, no definite genetic alteration specific for smoking was found [17]. An investigation of LOH and TP53 mutation by Inamura et al. has revealed combined effects of asbestos and cigarette smoking in the development of lung adenocarcinoma [22]. Moreover, Nymark et al. have reported that such a combination may lead to an increase in CNA, thus increasing the risk of cancer [7]. Taken together, these data suggest that, in the present case, cigarette smoking may have contributed more to the occurrence of CN gains in the PAC lesion than asbestos exposure.

One of the aims of this case study was to obtain data to indicate whether asbestos and cigarette smoking causes specific and characteristic gene alterations in these different kinds of target cells. Even in this single patient, the genetic events occurring in the two colliding tumors were quite different. This implies that tumor types arising from different cells, with same amount of asbestos exposure and cigarette smoke exposure and both in the thorax, would be differently impacted by each type of carcinogen exposure, though no conclusive evidence would be possible from this case study alone. A comprehensive genetic analysis of archival FFPE tissues would provide promising information to clarify this issue.

## Conclusion

We have compared CNA and somatic mutation in an exceedingly rare synchronous collision tumor consisting of MPM and PAC. Our CNA analysis showed that the frequencies of gain and loss were quite different between MPM and PAC. A comprehensive genetic analysis of these two different tumor types would provide data helpful for better understanding the thoracic carcinogenesis resulting from asbestos and cigarette smoking exposure.

## Consent

Written informed consent was obtained from the patient for publication of this case report and any accompanying images. A copy of the written consent form is available for review by the Editor-in-Chief of this Journal.

## Abbreviations

CA19-9: carbohydrate antigen 19-9
CEA: carcinoembryonic antigen
CN: copy number
CNA: copy number alteration
CT: computed tomography
CYFRA: cytokeratin fragment
FFPE: formalin-fixed paraffin-embedded
IHC: immunohistochemistry
LOH: loss of heterozygosity
MPM: malignant pleural mesothelioma
NGS: next-generation sequencing
NSE: neuron-specific enolase
PAC: primary pulmonary adenocarcinoma
Pro-GRP: pro-gastrin-releasing peptide
SCC: squamous cell carcinoma antigen

## References

[1] Murthy SS, Testa JR (1999). Asbestos, chromosomal deletions, and tumor suppressor gene alterations in human malignant mesothelioma. J Cell Physiol.

[2] Ivanov SV, Miller J, Lucito R, Tang C, Ivanova AV, Pei J (2009). Genomic events associated with progression of pleural malignant mesothelioma. Int J Cancer.

[3] Xio S, Li D, Vijg J, Sugarbaker DJ, Corson JM, Fletcher JA (1995). Codeletion of p15 and p16 in primary malignant mesothelioma. Oncogene.

[4] Cheng JQ, Jhanwar SC, Klein WM, Bell DW, Lee WC, Altomare DA (1994). p16 alterations and deletion mapping of 9p21-p22 in malignant mesothelioma. Cancer Res.

[5] Prins JB, Williamson KA, Kamp MM, Van Hezik EJ, Van der Kwast TH, Hagemeijer A (1998). The gene for the cyclin-dependent-kinase-4 inhibitor, CDKN2A, is preferentially deleted in malignant mesothelioma. Int J Cancer.

[6] Hirao T, Bueno R, Chen CJ, Gordon GJ, Heilig E, Kelsey KT (2002). Alterations of the p16(INK4) locus in human malignant mesothelial tumors. Carcinogenesis.

[7] Nymark P, Aavikko M, Mäkilä J, Ruosaari S, Hienonen-Kempas T, Wikman H (2013). Accumulation of genomic alterations in 2p16, 9q33.1 and 19p13 in lung tumours of asbestos-exposed patients. Mol Oncol.

[8] Björkqvist AM, Tammilehto L, Nordling S, Nurminen M, Anttila S, Mattson K (1998). Comparison of DNA copy number changes in malignant mesothelioma, adenocarcinoma and large-cell anaplastic carcinoma of the lung. Br J Cancer.

[9] Allen TC, Moran C (2006). Synchronous pulmonary carcinoma and pleural diffuse malignant mesothelioma. Arch Pathol Lab Med.

[10] Attanoos RL, Thomas DH, Gibbs AR (2003). Synchronous diffuse malignant mesothelioma and carcinomas in asbestos-exposed individuals. Histopathology.

[11] Sica G, Yoshizawa A, Sima CS, Azzoli CG, Downey RJ, Rusch VW (2010). A grading system of lung adenocarcinomas based on histologic pattern is predictive of disease recurrence in stage I tumors. Am J Surg Pathol.

[12] Ong M, Carreira S, Goodall J, Mateo J, Figueiredo I, Rodrigues DN (2014). Validation and utilisation of high-coverage next-generation sequencing to deliver the pharmacological audit trail. Br J Cancer.

[13] Bell DW, Jhanwar SC, Testa JR (1997). Multiple regions of allelic loss from chromosome arm 6q in malignant mesothelioma. Cancer Res.

[14] Takeda M, Kasai T, Enomoto Y, Takano M, Morita K, Kadota E (2012). Genomic gains and losses in malignant mesothelioma demonstrated by FISH analysis of paraffin-embedded tissues. J Clin Pathol.

[15] Sandberg AA, Bridge JA (2001). Updates on the cytogenetics and molecular genetics of bone and soft tissue tumors. Mesothelioma Cancer Genet Cytogenet.

[16] Andujar P, Wang J, Descatha A, Galateau-Sallé F, Abd-Alsamad I, Billon-Galland MA (2010). p16INK4A inactivation mechanisms in non-small-cell lung cancer patients occupationally exposed to asbestos. Lung Cancer.

[17] Huang YT, Lin X, Liu Y, Chirieac LR, McGovern R, Wain J (2011). Cigarette smoking increases copy number alterations in nonsmall-cell lung cancer. Proc Natl Acad Sci U S A.

[18] Karlsson A, Ringnér M, Lauss M, Botling J, Micke P, Planck M (2014). Genomic and transcriptional alterations in lung adenocarcinoma in relation to smoking history. Clin Cancer Res.

[19] Munafò MR, Timofeeva MN, Morris RW, Prieto-Merino D, Sattar N, Brennan P (2012). Association between genetic variants on chromosome 15q25 locus and objective measures of tobacco exposure. J Natl Cancer Inst.

[20] Kim HR, Kim DJ, Kang DR, Lee JG, Lim SM, Lee CY (2013). Fibroblast growth factor receptor 1 gene amplification is associated with poor survival and cigarette smoking dosage in patients with resected squamous cell lung cancer. J Clin Oncol.

[21] Lee M, Lee Y, Cho HJ, Hong J, Kwon SJ, Park CG (2011). Copy number variations of chromosome 17p13.1 might be linked to high risk of lung cancer in heavy smokers. Mol Biol Rep.

[22] Inamura K, Ninomiya H, Nomura K, Tsuchiya E, Satoh Y, Okumura S (2014). Combined effects of asbestos and cigarette smoke on the development of lung adenocarcinoma: different carcinogens may cause different genomic changes. Oncol Rep.

